# A little more conversation please? Qualitative study of researchers’ and patients’ interview accounts of training for patient and public involvement in clinical trials

**DOI:** 10.1186/s13063-015-0667-4

**Published:** 2015-04-27

**Authors:** Louise Dudley, Carrol Gamble, Alison Allam, Philip Bell, Deborah Buck, Heather Goodare, Bec Hanley, Jennifer Preston, Alison Walker, Paula Williamson, Bridget Young

**Affiliations:** Department of Biostatistics, University of Liverpool, 1st floor Duncan Building, Daulby Street, Liverpool, L69 3GA UK; TwoCan Associates, 59 Wickham Hill, Hurstpierpoint, Hassocks, BN6 9NR UK; Department of Women’s and Children’s Health, Institute of Translational Medicine (Child Health), Alder Hey Children’s NHS Foundation Trust, NIHR Clinical Research Network: Children, Coordinating Centre, University of Liverpool, Eaton Road, Liverpool, L12 2AP UK; Department of Psychological Sciences, University of Liverpool, Whelan Building, Liverpool, L69 3GB UK

**Keywords:** qualitative research, patient and public involvement, consumer involvement, stakeholder engagement, training, randomised controlled trials as topic

## Abstract

**Background:**

Training in patient and public involvement (PPI) is recommended, yet little is known about what training is needed. We explored researchers’ and PPI contributors’ accounts of PPI activity and training to inform the design of PPI training for both parties.

**Methods:**

We used semi-structured qualitative interviews with researchers (chief investigators and trial managers) and PPI contributors, accessed through a cohort of clinical trials, which had been funded between 2006 and 2010. An analysis of transcripts of audio-recorded interviews drew on the constant comparative method.

**Results:**

We interviewed 31 researchers and 17 PPI contributors from 28 trials. Most researchers could see some value in PPI training for researchers, although just under half had received such training themselves, and some had concerns about the purpose and evidence base for PPI training. PPI contributors were evenly split in their perceptions of whether researchers needed training in PPI. Few PPI contributors had themselves received training for their roles. Many informants across all groups felt that training PPI contributors was unnecessary because they already possessed the skills needed. Informants were also concerned that training would professionalise PPI contributors, limiting their ability to provide an authentic patient perspective. However, informants welcomed informal induction ‘conversations’ to help contributors understand their roles and support them in voicing their opinions. Informants believed that PPI contributors should be confident, motivated, intelligent, focussed on helping others and have relevant experience. Researchers looked for these qualities when selecting contributors, and spoke of how finding ‘the right’ contributor was more important than accessing ‘the right’ training.

**Conclusions:**

While informants were broadly receptive to PPI training for researchers, they expressed considerable reluctance to training PPI contributors. Providers of training will need to address these reservations. Our findings point to the importance of reconsidering how training is conceptualised, designed and promoted and of providing flexible, learning opportunities in ways that flow from researchers’ and contributors’ needs and preferences. We also identify some areas of training content and the need for further consideration to be given to the selection of PPI contributors and models for implementing PPI to ensure clinical trials benefit from a diversity of patient perspectives.

## Background

Patient and public involvement (PPI) in health research has been promoted within the United Kingdom at the highest levels for some time and it is increasingly encouraged internationally [1,2]. For research projects and programmes funded by some health and social care funders, PPI is mandatory, and for others, it is strongly encouraged, including in the United States, where it is known as stakeholder engagement, and in Australia, where it is termed consumer and community participation [2-6]. While the evidence on PPI activity in research is expanding, PPI training has received little research attention. Such training demands time and resources and it also has the potential to shape the future conceptualisation, implementation and impact of PPI in research. It therefore warrants research scrutiny.

INVOLVE [7], which is the UK-based advisory body on PPI in health and social care research, report that most PPI training courses have been developed within particular organisations or in the context of individual research projects. They define training broadly as any activity ‘that aims to help members of the public and researchers develop their knowledge, skills and experience to prepare them for public involvement in research’ [7]. An examination of training and educational provision of PPI in research confirms the diversity of aims, content and delivery of training. For example, education and training for PPI contributors ranges from a formally assessed and certificated year-long course on the discovery, testing and evaluation of medical products and technologies [8], to informal workshops that focus on helping contributors to identify suitable research roles and build confidence [9,10]. Examples of training for researchers are almost as variable, ranging from formal modules on the theory, policy and current practice of PPI within accredited Master’s courses [10,11], to single ‘awareness raising’ workshops on the aims and implementation of PPI in research [12].

While this diversity of training may be appropriate, it raises questions about how to ensure training is fit for the purpose. INVOLVE proposes that training be provided for both PPI contributors and researchers [7,13], tailored to the needs and roles of learners, delivered on an ongoing basis and that allows contributors and researchers to learn from each other [7]. These principles were drawn from consultations with over 30 stakeholders who had direct experience of PPI training either as providers or recipients. However, few details of the methods of consultation are available, and little is known about the perspectives of those researchers and PPI contributors who have not participated in training. A key consideration for any training is that it engages with the diversity of learners’ needs and is meaningful from their perspective [14]. Insights on how members of the research community perceive PPI training, whether or not they have had prior experience of such PPI training, will help to ensure its relevance and uptake.

This study aimed to provide such evidence in the context of PPI activity within randomised controlled trials (RCTs), a type of research thought particularly likely to benefit from PPI [15]. As part of a wider study of researchers’ and contributors’ perspectives on PPI, we explored their views and experiences of training in PPI and of related issues such as the selection and diversity of PPI contributors. We sampled informants from a cohort of RCTs and aimed to access individuals with a diverse range of perspectives.

## Methods

### Design

This qualitative study was part of the EPIC (Evidence base for Patient and Public Involvement in Clinical trials) project, which investigated PPI in a cohort of all RCTs in receipt of funding from the National Institute for Health Research (NIHR) Health Technology Assessment (HTA) programme between 2006 and 2010 [16,17], and for which relevant documents were available. Other components of EPIC included a review of PPI statements in the grant applications for each of the RCTs and surveys of chief investigators (CIs), PPI contributors and trial managers (TMs) on their views and experiences of PPI within these trials. For the qualitative study, we invited CIs, PPI contributors and TMs who had responded to the survey to be interviewed. While CIs and TMs might be anticipated to have similar perspectives, it was important to access both groups, as their roles in research are distinctive. Whereas TMs are researchers who usually only have contact with PPI contributors in the context of a trial, CIs often have clinical as well as research roles. Arising from their clinical roles, CIs might feel that they have knowledge of patients’ perspectives, which could influence how they value PPI. The interviews explored informants’ views and experiences of PPI and of training and support for PPI. In conjunction with this, we also explored how PPI contributors were selected into their roles. We describe the qualitative study methods in detail elsewhere [18].

### Ethics statement

We obtained ethical approval from the University of Liverpool Research Ethics Committee (Ref: RETH000489). All participants gave informed consent.

### Recruitment and sampling

We initially aimed to sample 25 trials and to use details provided on trial grant application forms to contact CIs. We initially sampled CIs for maximum diversity based on their survey responses to questions regarding their views on PPI, motivations for including PPI and who they involved, for example, patients or carers (see Table 1). However, we eventually invited almost all CIs who had responded to the survey and indicated an interest in being interviewed. We mainly accessed PPI contributors through CIs, by asking them to forward an email from the EPIC research team, which had invited PPI contributors to respond to the survey. We obtained contact details for TMs from clinical trial units, trial websites and protocols, or via CIs. As we were principally interested in the accounts of CIs and PPI contributors, we only invited the TMs for interview when a CI or PPI contributor from the same trial had been interviewed.

**Table 1:** **Survey responses used in initial sampling of chief investigators (CIs) for interviews and comparison of responses for interview subsample and survey sample**

**Question 1: In general, what is your personal view on patient and public involvement (PPI)?**	**CIs who were interviewed n(%)**	**Response distribution within CI survey n(%)**
PPI should always be included in a research study	12 (57)	42 (52)
PPI can be beneficial but is not always necessary	8 (38)	35 (43)
I am not convinced of the benefits of PPI	1 (5)	4 (5)
Question 2*: What motivated you to include PPI in your trial?		
I think including PPI is the right thing to do	15 (76)	51 (63)
I have previous experience of the benefits of PPI	12 (57)	45 (56)
PPI was a requirement for research funding	8 (38)	38 (47)
A PPI contributor offered their help	1 (5)	4 (5)
Other	2 (10)	9 (11)
Question 3*: Which PPI contributor/s did you involve?		
Patient	14 (67)	51 (63)
Carer	2 (10)	14 (17)
Parent	2 (10)	13 (16)
Charity member	10 (48)	24 (30)
Medical staff	4 (19)	11 (14)
Other	3 (14)	10,

*CIs could provide more than one response to questions 2 and 3.

### Interviews

LD, a psychologist, conducted audio-recorded semi-structured telephone interviews with informants between April 2013 and November 2013. LD explained that study data would be anonymised and kept confidential. Topic guided interviews were conversational to allow informants to freely voice their views and experiences of training for both researchers and PPI contributors, including the selection of PPI contributors and the provision of induction and support for their role. In order to minimise the risk of idealised accounts, LD adopted a neutral stance in her interviewing and used questions that were exploratory rather than ones that might be perceived as judgemental or interrogatory. We tailored the topic guides (summarised in Table 2) for each of the three informant groups (CI, PPI contributor and TMs) and periodically reviewed and developed the guides in the light of the ongoing data collection and analysis. Interviewing paralleled the analysis and continued until theoretical saturation [19] had been reached. Interviews were transcribed using an ‘efficient’ verbatim style that involved transcribing the content of informants’ accounts, rather than detailed features of speech such as sub-vocalisations and hesitations *etcetera*. All transcripts were checked for accuracy and anonymised.

**Table 2:** **Summary of interview topics covered**

**Researchers**	**Patient and public involvement (PPI) contributors**
**Expectations**	**Expectations**
Researchers understanding of PPI	Previous experience of being a PPI contributor
Experience of including PPI in research	Expectations for what working on the current trial would be like
Any initial goals or plans for PPI in current trial	
**What happened?**	**What happened?**
Stage of PPI implementation	How did they become involved in the trial?
Identifying and selecting PPI contributors	PPI contributor’s role
Roles of the PPI contributors	Relationship with research team
Overall experience of including PPI in the current trial	
**Impact**	**Impact**
Perceived contributions of PPI	Any differences made to the trial as a result of their input
Challenges of including PPI	Any benefits to themselves of being involved
	Any challenges of being involved
**Training and support**	**Training and support**
What training and support had been given to PPI contributors and views on this?	What training and support had they received and what are their views of training for PPI contributors?
Views and experiences of PPI training for researchers	What are their views on PPI training for researchers?

### Analysis

Analysis was informed by the principles of the constant comparative method [20,21], with elements of content analysis [22]. We drew on the concept of catalytic validity [23], which emphasises that findings should go beyond description to offer insights with potential to contribute to practice. Our approach was broadly interpretive; that is, as well as considering the manifest content of informants’ accounts, we looked at how participants described their perspectives and what was latent or deemphasised in their accounts, as well as what was emphasised [24]. While the emphasis in qualitative research is on being inductive, there were some deductive elements to our analysis; for example, we drew on current definitions of training [7] to inform the analysis. We used procedures to support rigour in qualitative research [25]. To ensure a contextualised analysis, we reviewed whole transcripts as well as focusing on particular sections. We analysed transcripts at the informant group level for evidence of CI, PPI contributor and TM views and experiences of training and support for PPI and how contributors were selected for the trials. Subsequently, we compared the accounts of the three informant groups to explore convergences and divergences between them. We also looked for deviant cases and used these to develop our analysis.

LD led the analysis, reading transcripts several times to develop open codes and liaising with BY, who also read multiple transcripts. LD and BY met regularly to compare interpretations of the data and review the ongoing analysis. Open coding took place at multiple levels from line-by-line coding of detailed descriptions to the general stance informants took towards PPI and training. These codes were grouped into categories and organised into a framework using NVivo 9 software. Categories were amended on an ongoing basis to ensure these reflected new data whilst accounting for deviant cases. To support our interpretations, we present illustrative extracts from the data. Extract codes were used to indicate the informant group CI, TM, PPI (1 or 2 if there were multiple PPI contributors on the same trial) and trial identification numbers.

## Results

### Sample

**Figure 1:**
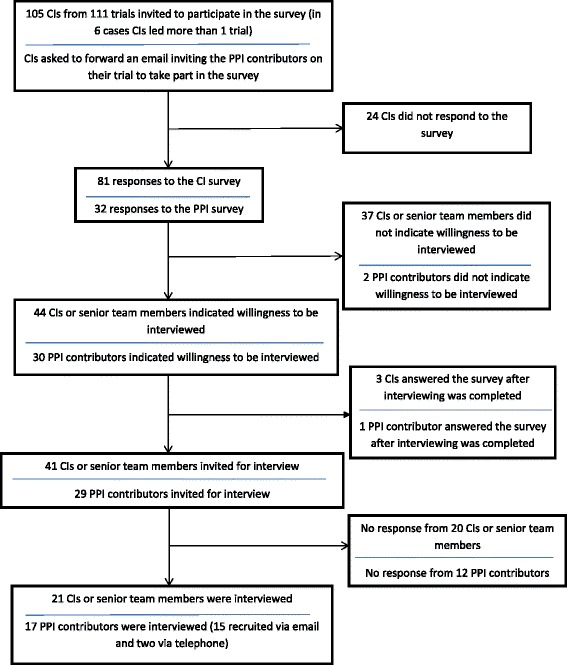
**Flow diagram illustrating chief investigator (CI) and patient and public involvement (PPI) contributor recruitment.** The figure provides a breakdown of CI and PPI contributor recruitment from the point at which potential informants were identified from those responding to the survey.

We recruited informants from 28 trials, which had been conducted in a variety of settings, and evaluated a range of interventions (Table 3). For 14 of the 28 trials, we interviewed one informant, for nine trials we interviewed two informants, for four trials we interviewed three informants, and for two trials we interviewed four informants. Figure 1 shows the recruitment of CIs and PPI contributors: 21/41 (51%) CIs and 17/29 (59%) PPI contributors were interviewed out of those invited. Regarding TMs, one trial did not have a TM at the time of our study, and we could not obtain contact details for three TMs. We invited TMs from the remaining 24 trials; of these, nine TMs did not respond, five declined and 10 (42%) were interviewed. PPI contributors who responded to the survey were mostly accessed via CIs, with one accessed via the chairperson of a trial steering committee. On average interviews lasted 45 minutes.

**Table 3:** **Informant interviewed, trial setting and intervention type**

**Trial**	**Chief investigator (CI) or senior team member interviewed?**	**Patient and public involvement (PI) interviewed?**	**Trial manager interviewed?**	Setting*	**Intervention**
**1**	Y	Y	N	Community	Education and exercise
**2**	Y	Y	N	Tertiary	Device
**3**	Y	Y	Y	Secondary	Education
**4**	Y	N	N	Tertiary	Drug
**5**	Y	N	Y	Secondary	Surgical
**6**	Y	Y	N	Secondary	Exercise
**7**	Y	Y	N	Primary	Community care
**8**	Y	Y (2 PPI contributors)	Y	Tertiary	Drug
**9**	Y	Y	Y	Secondary	Device
**10**	Y	N	N	Social care	Exercise
**11**	Y	Y (2 PPI contributors)	Y	Secondary	Surgical
**12**	Y	N	N	Secondary	Device
**13**	Y	N	Y	Secondary	Drug
**14**	Y	N	N	Secondary	Surgical
**15**	Y	Y	Y	Primary	Exercise
**16**	Y	N	N	Primary	Exercise
**17**	Y	N	N	Secondary	Surgical
**18**	Y	N	Y	Primary and secondary	Exercise and community care
**19**	Y	N	N	Primary	Other
**20**	Y	N	N	Emergency	Community care
**21**	Y	N	Y	Secondary	Device
**22**	N	Y	N	Secondary	Surgical
**23**	N	Y	N	Secondary	Device
**24**	N	Y	N	Tertiary	Drug
**25**	N	Y	N	Emergency	Surgical
**26**	N	Y	N	Secondary, Tertiary	Surgical
**27**	N	Y	Y	Emergency	Drug
**28**	N	Y	N	Secondary	Device

*A primary setting is the first point of consultation for a patient within the healthcare system, for example a general practitioner. A secondary setting involves healthcare provided by medical specialists who cannot be directly accessed by a patient, for example within a hospital outpatient clinic. A tertiary setting is specialist consultative healthcare, on referral from primary or secondary care and that has personnel and facilities for advanced investigation and treatment, for example a specialist cardiac unit.

### Induction and support: informal and implicit

We wanted to explore how informants conceptualised ‘induction’, and ‘support’ within the context of PPI without constraining their responses, so we avoided defining these terms during the interviews. Most informants characterised induction as a one-off informal ‘conversation’ between PPI contributors and researchers, rather than a formal or structured process. Inductions typically involved researchers explaining to PPI contributors what researchers ‘saw their input might be” (CI - 2) and ‘what the study was about’ (TM - 8), with PPI contributors being ‘given all the data, the explanation of what the trial was about’ (PPI - 6). Therefore, while both researchers and PPI contributors tended to describe ‘induction’ as an informal encounter, these were mostly one-way exchanges, with researchers being positioned as providers and PPI contributors as recipients. Neither party spoke of entering into a negotiation about what the role of the PPI contributors would entail.

Most informants reported that PPI contributors had received an induction. When PPI contributors had not received an induction, researchers and some PPI contributors explained that the PPI contributor had previous experience in a PPI role, and researchers had assumed an induction was superfluous in these cases. However, other PPI contributors who reported not having received an induction indicated that it would have been useful.

‘It would have been good to get a bit more of a sort of formal induction at the beginning about what the trial was, what my role was going to be’ (PPI - 9).

Similarly, researchers and PPI contributors described low key approaches to the support of PPI contributors, emphasising how PPI contributors knew they could contact researchers for support and advice if needed, although it was rare for them to do so.

‘We did not say ‘look we will give you support in this if you want it’, but I know that they had free and instant access to me. I would get emails and I would always respond, so even though it [support] might not have been explicitly stated, it was implicit’ (CI - 13).

‘We always organise all her train travel and that kind of thing […] she had my email; she had my phone number and things, and the same for the project administrator. So she always knew that she could contact us if she needed to’ (TM - 27).

‘I haven’t taken up much of it [support] but I know that the team, [name of CI] and his assistants have been there if I’ve needed to contact them, which I haven’t really, but I know that they’ve been there’ (PPI - 2).

Therefore, ‘offers’ of support for PPI contributors were largely implicit. In their interviews, researchers spoke of their readiness to provide support and contributors sensed that support was available if needed, although it seemed that both parties rarely discussed such support overtly during the course of PPI activities. Moreover, most informants saw support largely as synonymous with practical or logistical help, such as assistance with travel arrangements, rather than ongoing support to contributors in core aspects of their roles. From our analysis of CIs’ accounts, these roles included PPI contributors providing advice on patient recruitment and information materials, choice of outcome measures and the acceptability of the trial design. Although some PPI contributors felt unclear about their role, only one spoke of accessing support to address this.

‘I was in a learning process myself not knowing exactly what I could do, and I kept returning things to the professor saying, ‘I’ve been a bit pedantic here’ […] and she would say, ‘Pedantic is what I’m looking for, we need to have things pointed out to us that we may not have noticed’. So it was me learning how to approach the situation’ (PPI - 11-1).

As well as emphasising the informality of the induction process and the implicit nature of support, as we explain in the following sections, all three groups of informants spoke of their preference for informal ways of learning about PPI over more formal or structured training, particularly for PPI contributors.

### Training

Mirroring their accounts of induction, informants often drew a distinction between formal training and informal ‘conversations’ about the trial. Most informants tended to conceptualise ‘training’ as ‘formal’, structured courses. A summary of the training needs that were identified by researchers and PPI contributors can be found in Table 4.

**Table 4:** **Training needs identified by researchers and patient and public involvement (PPI) contributors**

	**Training for researchers**	**Training for PPI contributors**
**Training needs reported by researchers**	• Guidance on how and when to involve PPI contributors	• General research methods or design
	• How to get the most out of contributors	• Role expectations
		• What happens in meetings
	• What is expected of contributors	• Confidence to speak in meetings
	• How PPI benefits research	
	• Guidance on payment	
**Training needs reported by PPI contributors**	• Avoiding jargon	• How PPI and research works
	• Role expectations	• How ethics and funding works
	• How to engage PPI contributors	• Being able to ask questions/ confidence to speak up
		• Role expectations

#### Researchers on their own training: useful to a point

Of the 31 researchers interviewed, 18 had not had any form of training in PPI, while 13 had accessed training or attended an informal talk or workshop on PPI. These had covered topics such as ‘how PPI should work’, how to incorporate PPI into research, how to run focus groups and how to identify and engage PPI contributors. All researchers who had participated in these activities had found them useful in discovering how to do PPI, what had worked for others and how PPI can benefit research. However, one CI commented that the formal training he had received was too focussed on ‘how PPI should work’ and that insufficient emphasis had been given to ‘the practicalities of how to involve your patients’ (CI - 5).

Of the 18 researchers who had not received training, nine indicated that they would like to receive training and nine expressed reluctance. CIs who wanted training explained that they would like guidance on how and when to involve PPI contributors, how to optimise their input, what is expected of PPI contributors, how PPI benefits research and guidance on payment for PPI. Additionally, one TM wanted to learn about the wider research community’s expectations regarding the implementation of PPI:

‘A little bit more on how we can work together, patients and researchers, and how we can benefit from having a strong PPI involvement in this study […] I’d like to learn a bit more on how things should be done or how they are expected to be done’ (TM - 21).

Of the 21 CIs, 6 had no experience of PPI prior to the current trial. Researchers who did not want training commented that they already knew ‘how to do PPI’ usually because they had learnt about it ‘through experience rather through any particular formal training’ (CI - 5). While researchers, particularly CIs, generally described one of the main challenges of PPI to be finding ‘suitable’ people, they did not identify this as a training need. Nor did we find any evidence that researchers’ views on training were influenced by their views on the value of PPI. For example, some researchers who were sceptical about the value of PPI felt that training would be useful for certain topics. Conversely, some researchers who spoke of PPI as ‘really important’ or ‘crucial’ were reluctant to receive training because they could not envisage what topics the training would cover, or felt that there was insufficient knowledge of PPI in order to inform a training course.

‘Hopefully your research project will come out with sort of clearer guidelines about when and where PPI input is useful, because I’m never quite sure myself […] I wouldn’t be keen on any training at the moment’ (CI - 15).

#### Patient and public involvement contributors on training for researchers: useful to address specific difficulties

Of the 17 PPI contributors, nine felt that researchers should receive training in PPI. Contributors pointed to the use of jargon as one of the main challenges they faced. Reflecting this, they indicated that researchers could benefit from training on the importance of using plain English. Some PPI contributors also pointed to the lack of role clarity as a challenge they faced and reflecting this, they spoke of understanding the public mindset, contributors’ roles and how to engage with them as priority areas for researcher training.

‘I’d be told what […] the expectations of me were in the team and what are the things I was supposed to do in the team, and they [researchers] would have that explained to them as well […] It would just be making sure that people understood my role’ (PPI - 22).

Other PPI contributors felt that training researchers was unnecessary, particularly as they saw CIs as individuals who were used to ‘dealing with people’ and expected researchers to have acquired the requisite knowledge and experience or ‘at a level of um ability, shall we say, that shouldn’t need much training’ (PPI - 11). A few PPI contributors also felt that such interpersonal abilities could not ‘be taught, it’s a question of interface and interaction and experience with one another’ (PPI - 7).

There were indications that PPI contributors’ views on training for researchers may be linked to their previous PPI experience. Of the nine PPI contributors who felt that researchers should receive training, six had little or no experience of being a PPI contributor. Conversely, of the five PPI contributors who felt that researchers did not need training, four had previous experience of being a PPI contributor.

#### Patient and public involvement contributors on their own training: largely unnecessary and potentially detrimental

Of the 17 PPI contributors, only three had received training for their role within the EPIC cohort trial. They indicated that this training had focussed on research processes including, ‘basic appraisal of clinical papers’, ‘general research training’, and what research is about and good clinical practice, rather than the roles of PPI contributors. All three contributors described the training as useful, although the contributor whose training had covered critical assessment of research papers focussed on how it had informed her paid employment as a nurse rather than her PPI role.

‘I learned a lot […] I’m actually able [to] apply it to other aspects of my work now because if we’re looking at products in the area I work in, I can look at the clinical papers attached to them and understand more how to read them, so it has been a help to me’ (PPI - 2).

The remaining 14 PPI contributors did not report having received any training. Of all 17 PPI contributors, 15 indicated that they did not want or need training, with most explaining that a conversation at the beginning of their involvement to clarify their role would be sufficient. Our wider analyses had identified three main roles for contributors: oversight, managerial and responsive. Both PPI contributors and researchers acknowledged that a contributor’s training needs depended on the particular type of role they had. In oversight roles contributors’ activities were formal and often entailed being the sole PPI member on a trial steering or data monitoring committees. Managerial roles were also formal and usually entailed one PPI contributor acting as a co-investigator or member of the trial management group. Responsive PPI roles were typically more informal than the other two types, with contributors being approached for advice on an ‘as required’ basis. All the contributors in our study had oversight or managerial roles within the EPIC cohort trials. Some spoke of not needing training because they had already acquired the necessary skills for these roles through their employment or previous experience as a PPI contributor. For example, a contributor who described himself as having worked as a ‘senior manager in industry’, and whose description of his role within the trial resembled that of a manager, commented that training was unnecessary because he was:

‘Well used to running meetings and keep people on track […] I’m used to actually making decisions and coming up with proper ways of getting things done’ (PPI - 22).

His account stood in contrast to another contributor who saw her role as more limited.

‘I can use a computer and I can use email and things like that because of my job, […] but as far as training is concerned, because my role is absolutely not to run the trial or anything […] I’m not sure that training per se is necessary’ (PPI - 11).

Despite the differing perceptions of their roles, both contributors talked of how they already possessed the skills their roles required and neither could therefore see a need for training. Some PPI contributors gave passing mention to how training may be useful for ‘other’ contributors who had less experience.

‘I’ve had eight years of being a non-exec director, so I know what is expected […] so no, I didn’t need it [training]. I would suggest other PPI members […] would need some training and familiarisation in how it works’ (PPI - 26).

Two contributors were also concerned that training could result in PPI contributors learning ‘too much’, which would be detrimental to providing a patient perspective. For example, training could encourage contributors to look at issues ‘from a clinician’s point of view […] once you’ve learnt too much I don’t believe that you’re a lay person’ (PPI - 23/24). Interestingly, these PPI contributors spoke of such professionalisation in general or hypothetical terms, rather than as something they struggled with personally.

#### Researchers on training for patient and public involvement contributors: better to select than train

Of the 31 researchers interviewed, 6 indicated that training would be of value for PPI contributors on their trial, 6 did not express an opinion and 19 felt that training was unnecessary. Like the contributors, researchers in the latter group regarded PPI contributors as already possessing the skills and experience that their roles required.

‘I don’t think that she [PPI contributor] was in any way reserved about contributing and I think she understood the role on the trial steering committee […] because she had had a role of representing patients before’ (CI - 4).

Informants’ accounts of training were closely bound up with their accounts of how to select PPI contributors, and both researchers and PPI contributors implied that, rather than training PPI contributors, it was often better to select individuals who already possessed the attributes necessary for their role:

‘It can take a lot of time to bring people up to speed with the principle of trial design. And I’m not saying that’s not necessarily a good thing to do but if somebody’s already got that experience and knowledge you’ve already overcome quite a big hurdle’ (CI - 9).

Six researchers echoed the concerns of the PPI contributors who thought that training could over professionalise PPI contributors and therefore be detrimental to their role:

‘If you train them then I think they’re probably aware of more of what should be happening and they won’t have such an objective view, whereas if they’re coming at it almost totally fresh then they have more of the perspective of if a patient received this information’ (TM - 18).

‘What you really want from them is for them to be kind of impartial and […] to really represent what the patients and the public think. So if they are too kind of clued up on research they might not actually be representative of our target audience’ (TM - 13).

A few researchers also struggled to identify what the content of training would be for PPI contributors: ‘I would find it difficult to know what you could train people in’ (CI - 11). Researchers who felt that training would be beneficial for contributors on the trial, pointed to general research training such as research methods and the role of PPI contributors as suitable topics. While researchers generally tended to emphasise that the particular PPI contributors on the EPIC cohort trial did not need training, some pointed to circumstances when training might be useful. For example, contributors who were ‘less experienced’ could benefit from training, particularly if they were to have oversight or managerial roles.

‘Making sure that the person who is the PPI rep is comfortable and confident to ask questions and to query things […] that’s maybe when training actually for the PPI, now I think about it, might be really important’ (CI - 6).

‘If you’re expecting them to attend the trial steering committee, if they’ve not done that type of thing before, then I think some information and training […] around the topics that are going to be come up’ (TM - 18).

### Need for ‘professional’ and ‘lay’ contributors

According to our informants, it was not just training that could lead to the professionalisation of PPI contributors, cumulative experience in a PPI role could also do this. Both researchers and PPI contributors distinguished between “lay” and “professional” PPI contributors, although the latter were not necessarily individuals with professional employment backgrounds. Researchers in particular described professional contributors as people who ‘went around doing PPI’, implying that such individuals had a level of experience and knowledge that set them apart from other patients, whereas lay contributors were ‘just […] people with the experience of whatever it is you’re researching’ (CI −3). Professional contributors were believed to get ‘less and less like the population the more engaged they become’ (CI - 16), and increasingly influenced by the ‘researcher mind set’ and unable to contribute an authentic patient perspective. Conversely, researchers acknowledged how difficulties could arise when PPI contributors were ‘naïve’ to their role in research. One CI spoke of a difficultly she had experienced on a previous trial with a PPI contributor who had been ‘preoccupied by getting their personal healthcare improved’ (CI - 7) and others commented on how it was helpful to have contributors who understood the research process and the constraints on researchers.

‘When it works it works probably because we’ve got people who are able to understand what we want of them and are able to be quite articulate and succinct and able to separate out their own stuff from the problem on the table’ (CI - 20).

Some researchers were torn between wanting the benefits that professional contributors brought and worrying that they were hardly ‘representative’ of target participants. Others spoke of how this tension could be resolved by involving both professional and lay PPI contributors in trials. Whereas professional PPI contributors were believed to be suited to managerial or oversight roles, lay PPI contributors were felt to be suited to responsive roles, which required a ‘true’ patient perspective and where it was possible to ‘come along and be yourself’.

‘I was a little bit surprised to find that there were these sort of professional PPI reps. I think it is good because they come with an understanding of research, but I think that’s where it’s really important that you have a mix of people that you’re getting views from, because I think if maybe people are too research-savvy, they’re only going to think like researchers and they’re not actually going to give us a real patient perspective’ (CI - 6).

### Selecting patient and public involvement contributors

Most researchers had identified PPI contributors through a charity or patient organisation or indicated that the PPI contributor was previously known to them, either through earlier PPI work or as participants in a previous trial. Two CIs had PPI contributors who were their own patients. Sometimes CIs had sought or received recommendations regarding the suitability of a potential PPI contributor from other researchers, PPI contributors, patient organisations or personal contacts. Only one CI reported having sent an advert out through a patient organisation inviting people to volunteer for the role of PPI contributor.

As noted above, informants from all groups described the importance of selecting PPI contributors to suit their role. As we illustrate in the list below of attributes and qualities that informants thought a PPI contributor should posses, all informant groups emphasised the importance of selecting contributors who could be confident and active in meetings if they were to have oversight and managerial roles, and spoke of how PPI contributors should be motivated and have interests in the research or clinical area. Although some informants, as we note above, commented that it was important for PPI contributors to have previous experience of a PPI role, the lack of such experience could be compensated for in other ways, for example, if contributors shared characteristics or experiences in common with the participants to be targeted for a particular trial or had an educational or employment background seen as relevant to their role.

### Attributes and qualities that informants thought patient and public involvement (PPI) contributors should possess

- **Confidence**
  - ‘You want them to be confident and capable of presenting their view. So you don’t want somebody who’s going to be shy. You don’t want somebody who’s going to be easily intimidated’. (CI - 9)
    ‘They’ve [PPI contributors] got to be a bit of a confident person to speak up in these kinds of meetings. You’ve got lots of clinicians, they’re talking about lots of things that the patient probably doesn’t understand’. (TM - 5)
    ‘You do have to be confident to make your point. So you’re working with clinicians, they talk quite technical […] being happy to make your point can be challenging, but I do it and I’m comfortable doing it’. (PPI - 26)

- **Motivation and commitment**
  - ‘Finding the right person who has the time and the commitment and the interest’. (CI - 20)
    ‘Someone who’s interested in research […] who’s willing to commit […] So if we wanted someone on one of our steering committees they would have to be able to commit to a certain number of meetings a year and be happy to review documents in time for meetings’. (TM - 9)
    ‘They [PPI contributors] should be passionate people that really believe that they, they want to try and make a difference’. (PPI - 7)

- **Focus on the ‘greater good’**
  - ‘My PPI people were really good because they never, ever […] brought it back down to themselves all the time […] But in another study that I’ve got, we have had that and it’s become quite draining to everybody because the PPI […] person has become very much preoccupied by getting their personal healthcare improved’. (CI - 7)
    ‘An ability to see beyond their own particular experience and maybe draw on the experience of peers, others in the field’. (TM - 3)
    ‘They should be people who can look outside of their immediate needs to the greater good […] if I was particularly concerned about my condition then it could override everything in terms of the trial. I might want to steer it towards something I’m particularly keen to have resolved or sorted. So you need to have someone who sees the trial, and understands the concept in the broadest sense. And that the benefits may not indeed help them’. (PPI - 26)

- **Previous PPI experience**
  - ‘It’s useful [to have contributors with previous PPI experience] because they have an understanding both of the general world, if you can call it that, and also of the research field, so, and of what their researchers are expecting […] It probably does help if they have had some experience before, but it’s not always necessary’. (CI - 1)
    ‘We had people who, who had been [contributors] on other randomised controlled trials so they, they knew what we were getting at but […] you’re bound to get somebody who… for whom it’s, it’s new but as long as […] you’re not asking them to think about something that they can’t possibly have any familiarity with, then I think you can overcome the lack of methodological experience’. (CI - 16)

- **Experience of the target condition, patient group or of interventions similar to those being investigated**
  - ‘One of the most important things is that they represent the type of person that the study will be directed at. There’s no point going for a fifty-year-old guy when your study is about [condition] and you’re going to be recruiting people who are under 24’. (CI - 19)
    ‘We often recruit more broadly than just patients now. So it’s carers and people who work with frail elderly patients who often have greater time and as much insight as the patient themselves in many ways’. (CI - 5)
    ‘It’s important that they’ve […] had experience of having the type of operation that you’re investigating, or the disease area that you’re investigating, just so that they’ve got a better perspective of what is important to the patient’. (TM - 11)
    ‘[PPI contributors should be] people that have experience of the condition […] it should be condition-specific’. (PPI - 7)

- **Intelligence and education**
  - ‘It’s got to be somebody who, is sort of, I don’t know if intelligent is the right word, but who has a lot of common sense basically. Obviously [they] do need to be intelligent and be able to read moderately complex stuff and understand fairly complex things. You don’t want somebody who’s not very bright’. (CI - 71201)
    ‘You’ve got to be quite bright. I think some of these documents are quite dense, so I think that that’s important’. (PPI - 11)
    ‘You need fairly reasonably academic and, or professional qualifications to do it, so it’s not for the faint hearted […] I’ve got a university degree, and I’ve written one or two books […] others might be bewildered by it’ (PPI - 15).

## Discussion

### Summary

Informants had some reservations about the need for training in PPI, particularly in relation to training PPI contributors. Very few contributors had received training for their roles, and many were reluctant to engage in it. Researchers shared this lack of enthusiasm for training PPI contributors. However, informants did welcome informal induction ‘conversations’ to help contributors to understand their roles. Beyond this, they tended to see training PPI contributors as redundant because, by selection, they already possessed the necessary attributes. Informants were also concerned that training and cumulative experience in PPI roles could lead to the professionalisation of contributors, limiting their ability to ‘represent’ the patient perspective. Researchers described a tension between the need for contributors who could offer an authentic patient perspective and a need for contributors who could function in oversight and managerial roles (for example as members of trial steering and managerial groups, respectively). Some researchers commented that this tension could be resolved by selecting particular PPI contributors for particular roles. Rather than training contributors, researchers favoured using their networks and others’ recommendations to select individuals who already possessed attributes perceived as important for the role. It could be that they felt it was more efficient to select contributors than train them or believed that the particular qualities required of contributors were not readily amenable to development via training. Informants were more receptive to training researchers in PPI. In contrast to PPI contributors, most researchers had either received training or indicated that they would find it helpful. Nevertheless, a sizable minority did not think training would be helpful because they had learnt about PPI ‘on the job’ or felt that there was insufficient evidence to inform training. Contributors also saw a fairly limited role for training researchers in PPI, although they did point to the use of plain English and clarity about PPI contributor roles as specific areas in which researchers could benefit from training. For reasons which we outline below, despite the reticence towards training that we found, we caution against interpreting our findings as evidence to abandon PPI training. However, the findings do point to ways in which the current conceptualisation, design, promotion and delivery of training could be enhanced.

### Previous research and implications

There have been few previous empirical studies of PPI training either for researchers or PPI contributors, although training has generally been recommended for both groups [7,26,27]. While not specific to clinical trials, previous research has highlighted potential areas for training for both PPI contributors, such as the aims, design and methods of research, supporting PPI activities and facilitating partnerships within the research team [27]. In showing that the appetite for PPI training is limited, particularly for PPI contributors, our findings diverge from previous research, which found that researchers and PPI contributors were in favour of training [27,28]. This divergence may reflect differences in sampling between previous work and ours, and particularly our focus on clinical trials. Informants in our study knew each other and their reluctance towards training could reflect a concern to avoid implying criticism of their colleagues, as well as themselves. Nevertheless, it is worth noting that previous research has tended to seek informants’ views on training in general, whereas we sought informants’ views about training both for themselves and for the researcher or PPI contributor with whom they worked. Interestingly, we found informants were more receptive to training when speaking of ‘others’ outside their trials. Some of our informants also struggled to imagine what the content of training might be. Therefore, at the very least, our findings indicate that those providing PPI training and wishing to improve its uptake need to articulate what both researchers and contributors can expect to gain from training.

Researchers also questioned what evidence was available on ‘how to do PPI’ and thereby to inform training. Such evidence is currently limited, at least in part because the promotion of PPI activity in research, rather than its evaluation, has been the focus of much of the published literature [15]. In separate articles from the EPIC study, we have described researchers’ and PPI contributors’ accounts of how the impact of PPI can be enhanced within trials (LD et al. unpublished work) and the lessons that researchers and PPI contributors learnt through their PPI activities [18]. This and similar evidence from those at the frontline of PPI practice could contribute to the evidence base for training and inform its design and delivery.

Some informants, particularly researchers, were concerned that training could hamper contributors’ ability to provide a patient perspective. While such concerns need to be taken seriously, it is possible to envisage ways that training could support rather than detract from this ability. Indeed, how to maintain a patient perspective could be an explicit focus of education for PPI. More fundamentally, our findings have implications for how PPI training is conceptualised. The pronounced reluctance that we identified regarding training for PPI contributors, and informants’ preferences for ‘conversation’ over ‘training’, align with the emphasis informants gave to establishing good relationships between the PPI contributors and the research team (LD et al. unpublished work). If a priority is to establish relationships, it is perhaps not surprising that informants saw engaging in mutual conversation as a more natural way to develop good working relationships with each other than engaging in training. The term training is often associated with the acquisition of technical or practical skills, and it may be that alternative types of educational provision such as action learning sets [29,30] and coaching [31] are more suited to learning about PPI. Researchers and contributors may welcome such changes to educational provision for PPI. However, given the rather implicit and one-way nature of induction for PPI contributors as it was seemingly practised in the EPIC trials, our findings also point to the need for a more structured approach to induction, as well as the need to provide opportunities for contributors to negotiate their roles with researchers.

Our study also indicates the training needs that researchers and PPI contributors identified (summarised in Table 3). While these are similar to training needs that have been previously identified [27], it is notable that informants in our study tended to speak of training for PPI contributors in terms of learning ‘how to do research’ whereas training for researchers was spoken of more broadly and included learning ‘how to do PPI’. This raises questions about what should be the aims of PPI training for both contributors and researchers, and about how these aims are communicated. Given that some PPI contributors lacked clarity regarding their roles, provision that helps both researchers and PPI contributors to learn about ‘how to do PPI’ would be beneficial. The perception that training for PPI contributors focuses on ‘how to do research’ may be the source of some of the reluctance that surrounded training; how much contributors need to know about research in order to be effective in their roles requires serious consideration. Regarding delivery, our informants also described training as something that was delivered separately for PPI contributors and researchers. However, reflecting their emphasis on the need to develop good relationships and mutual understanding of roles, our findings support previous suggestions that training should allow contributors and researchers to learn from each other [7]. Indeed, consideration might be given to training PPI contributors and researchers side-by-side.

As well as posing some questions for PPI training, our findings also raise questions about the selection of PPI contributors. Researchers indicated how they worked to select PPI contributors with certain attributes, yet recognised that this brought into question the claim that such contributors could voice the patient perspective. Individuals who are educated and articulate have been found to be particularly likely to volunteer as PPI contributors [32]. As many of our informants indicated, such PPI contributors may not be in a position to speak for those from less advantaged backgrounds. There is a danger that when combined with reluctance towards training contributors, PPI contributor selection practices, if reproduced across many studies, could gradually mould research to the preferences of advantaged groups. There are also indications that PPI contributors with higher levels of education may be prone to over identify with the perspectives of researchers rather than challenging them [32]. Therefore, while our informants did not themselves identify the selection of PPI contributors as a training need, our findings indicate that it is an area in which researchers could benefit from educational provision. Informants in our study did, nevertheless, point to the importance of involving both professional and lay PPI contributors, the former in managerial or oversight roles, and the latter in responsive roles via patient advisory panels. Such mixed models of PPI could help to ameliorate these difficulties and address the multiple functions required of PPI within clinical trials.

### Strengths and limitations

To our knowledge, this the first study to interview researchers and PPI contributors who had no experience of training as well as those who had such experience. Our study provides insights about how PPI training could be developed to enhance its relevance and uptake for both contributors and researchers. By exploring training needs in the context of informants’ experiences of PPI activity, rather than focussing narrowly on training, our study has also identified some potential topics for training beyond those articulated by our informants. The sample included informants who had previous experience of PPI as well as those without such experience. While there were indications that those with previous experience of PPI would have little to gain from training, in interpreting informants’ accounts, it seems relevant to note that research in a variety of educational contexts has indicated that experience alone is not necessarily the best teacher [33,34] and that individuals are not necessarily accurate in assessing their own knowledge and skills [35]. This raises an important limitation: our study was not intended to formally assess informants’ knowledge and understanding of PPI or their skills in implementing PPI. Such assessment would almost certainly identify additional training needs. While the perceptions of researchers and contributors are important to inform future educational provision for PPI, assessment of informants’ PPI knowledge and skills would be valuable to address wider questions about the effectiveness and value of PPI training. Our study was retrospective and it is possible that prospective work would identify further training needs. Also, informants’ accounts of training may have been shaped by their perceptions of the success or otherwise of the trial. All the interviewed PPI contributors had been involved in managerial or oversight roles, and further research is needed on training for those in responsive roles. As we note above, informants’ reluctance regarding training for themselves and their colleagues may have been a way to avoid implying criticism of both parties. However, as we report elsewhere [18], both researchers and contributors gave critical accounts of aspects of their own and their colleagues’ approach to PPI, and it seems unlikely that reticence to criticise would manifest only in relation to training. The reluctance that we found may also reflect a sense of being too busy to participate in training, which might, in turn, be indicative of a propensity to devalue PPI [23], although we found no evidence that the attitudes of researchers to PPI were linked to their views of training. PPI contributors also expressed reluctance towards training, yet it seems implausible that this could be attributed to them holding unfavourable attitudes to PPI.

Our sample was drawn from a wider cohort study and survey, and this enabled us to provide more information about our sample than is perhaps typical for qualitative studies. Although we aimed to purposively sample informants to gain a diversity of views, we ended up inviting almost all of those who indicated willingness to be interviewed. Compared to the wider sample of CIs in our survey, the survey responses of the sub-sample that we interviewed indicated that they were slightly more favourably inclined to PPI than those CIs who were not interviewed. However, it would be epistemologically inappropriate to assume that survey responses can be taken as a ‘true’ point from which to assess the adequacy of our sample [36]. As we also report elsewhere (CG et al. currently unpublished work), regardless of their survey responses, CIs in the interviewed sample expressed a diversity of views about PPI and some were sceptical about its value. Finally, with one exception, our access to PPI contributors was limited to those whose contact details were provided by CIs. The relationships between interviewed contributors and researchers might have been more favourable than those who were not interviewed. It is therefore possible that contributors and researchers developed a shared mindset that manifested as reluctance towards training. Nevertheless, members of both groups gave critical accounts of the other groups’ approaches to PPI so it seems unlikely that either group struggled to articulate their own perspectives regarding training.

## Conclusions

While informants believed that training researchers in PPI was useful to an extent, they saw little or no need to provide training for PPI contributors. They regarded such training as unnecessary and as limiting the ability of PPI contributors to offer an authentic patient perspective. Informants regarded informal conversational approaches to helping contributors learn about their roles within trials as more appropriate than providing training. Our study was not intended to address questions about the effectiveness of training and should not be interpreted as providing evidence that educational provision for PPI be abandoned. Rather, in pointing to the need for further consideration to be given to how training is conceptualised, designed, promoted and delivered, the findings will be useful to those who wish to enhance the uptake and relevance of PPI training. The findings also indicate the value of providing flexible, informal induction and learning opportunities in ways that flow from researchers’ and contributors’ needs and preferences. However, instead of providing training, we found that researchers preferred to select PPI contributors who possessed certain qualities, despite recognising that this raised questions about how well placed such individuals were to speak for those from very different backgrounds to their own. In order to ensure that clinical trials benefit from a diversity of patient perspectives, the research community needs to give further consideration to processes for selecting PPI contributors, to the potential role of training in widening the pool of available PPI contributors and to the value of different models of implementing PPI.

## Abbreviations

CI: chief investigator
EPIC: Evidence base for Patient and public Involvement in Clinical trials
HTA: Health Technology Assessment
NIHR: National Institute for Health Research
PPI: patient and public involvement
RCT: randomised controlled trial
TM: trial manager.
